# Effects of water depth on GBD associated with total dissolved gas supersaturation in Chinese sucker (*Myxocyprinus asiaticus*) in upper Yangtze River

**DOI:** 10.1038/s41598-019-42971-8

**Published:** 2019-05-02

**Authors:** Lu Cao, Yong Li, Ruidong An, Yuanming Wang, Kefeng Li, Kurt Buchmann

**Affiliations:** 1 School of Port and Transportation Engineering, Zhejiang Ocean University, Zhoushan, 316000 Zhejiang China; 2 State Key Laboratory of Hydraulics and Mountain River Engineering, Sichuan University, Chengdu, 610065 China; 3 Laboratory of Aquatic Pathobiology, Department of Veterinary and Animal Sciences, Faculty of Health and Medical Sciences, University of Copenhagen, DK-1870 Frederiksberg C, Denmark

**Keywords:** Ichthyology, Freshwater ecology

## Abstract

Spillway water falling from hydroelectric power plant dams in the upper Yangtze River creates a high pressure in plunge pools below the dams allowing gasses to be dissolved at high rates. The resulting supersaturation persists many miles downstream the dam which may elicit mortality in river fishes associated with gas bubble disease (GBD). We have in a two-year study (2014–15) evaluated the effect of water depth on development of GBD in an endemic and endangered fish species, the Chinese sucker *Myxocyprinus asiaticus*, 24 km downstream of Xiangjaiba dam. Mortality and incidence of GBD were recorded and it was seen that water depth and survival time/GBD development was positively correlated. The physiological mechanisms explaining increased resistance to GBD with increased water depths (and thereby higher hydrostatic pressure) are discussed. The results may be applied in future management of fish resources in order to protect endangered endemic fishes in rivers affected by dam constructions.

## Introduction

A range of vulnerable fish species occupy the Yangtze River basins which is the main center for existing and planned hydroelectric power plants in China but the ecological impact of this type of energy production has become increasingly clear during the last decades. Development of gas bubble disease (GBD) and increased mortality in endemic fish populations has been observed to follow power plant discharge periods associated with gas super-saturation of river water^1,2^. Spillway discharges of river water from dam constructions end up in a plunge pool below the dam resulting in a huge gas pressure generating gas-supersaturated water often recorded as total dissolved gas supersaturation (TDGS)^3^. De-gassing of gas-supersaturated water occurs slowly whereby supersaturation persists for many miles downstream the dam^4^. The problem was previously documented in American studies showing that supersaturation can persist up to 150 km downstream the dam production site^5^. In North America high supersaturation levels were reported with TDGS levels of 110% (Midwestern reservoir of America)^6^, 125% (Columbia and Clark Fork Rivers)^7,8^, 130% (Ice Harbor Dam in the Snake River)^9^ and 140% (downstream the Keenleyside Dam and Arrow Dam (Columbia River). In China, the TDGS levels downstream dam constructions associated with the Three Gorges, Ertan, Gongzui and Tongjiezi have commonly reached 130%^10^ and in 2007 the maximum TDGS level was reported as high as 143%^11^. Exposure of aquatic organisms, including fish, to gas-supersaturated water may affect the swimming performance^12^ and elicit gas bubble disease (GBD)^13^ associated with development of emboli (in the vascular system) and emphysema (in tissues). GBD has previously been associated with marked fish mortality, as reported from Norway^14^ and USA^15^, and in 2014, a mass mortality affecting 40 metric tonnes of various fish species was recorded below the upper Yangtze River’s Xiangjiaba Dam reservoir (China), probably due to TDGS effects^16^. However, it has been suggested that some fish species including steelhead trout (*Oncorhynchus mykiss*), brown trout (*Salmo trutta*), perch (*Perca*) and chinook salmon (*Oncorhynchus tshawytscha*) perform better in deeper waters levels when exposed to TDGS^14,17–19^ and this may apply also for the Yangtze River fishes. The Chinese sucker (*M. asiaticus*) is one of the national second-class protected animals in China occuring in the upper Yangtze and Min River^20,21^ and it is noteworthy that populations of this fish species has suffered increased mortality following the operation start of the Xiangjiaba and Xiluodu dam plants in the upper Yangtze River. It is therefore relevant to investigate the effect of TDGS on Chinese sucker and explore if water depth may alleviate any adverse effects. The present study reports on the effects of different water depths on GBD and fish mortality. Results may be used to develop a strategy for the future protection of fisheries in the Yangtze River.

## Materials and Methods

### Ethical statement

The animal study proposal was approved by the Ethics Committee for Animal Experiments of Sichuan University. All experimental procedures were performed in accordance with the Regulations for the Administration of Affairs Concerning Experimental Animals approved by the State Council of the People’s Republic of China.

### Fish

A total of 330 Chinese suckers were used for the study conducted over two years. One hundred seventy-nine fish (mean ± SE length 8.8 ± 0.12 cm, and mean ± SE weight 18.5 ± 0.70 g) and one hundred fifty-one fish (mean ± SE length 6.63 ± 0.01 cm, and mean ± SE weight 8.90 ± 0.03 g) were supplied by the local fisheries research institute in August 2014 and September 2015, respectively. Fish were allocated to net cages placed at different water depths according to Table 1.

**Table 1:** No. and size (length and weight) of experimental fish in different groups.

Year	Conditions	N	Length (cm)	Weight (g)
2014	Surface (0–0.3 m)	30	9.38 ± 0.06	20.90 ± 0.38
		30	8.21 ± 0.04	14.93 ± 0.20
	0–1 m	20	9.02 ± 0.09	19.05 ± 0.44
		20	10.99 ± 0.07	32.15 ± 0.46
	1–2 m	20	8.07 ± 0.04	14.40 ± 0.21
		20	8.82 ± 0.08	18.65 ± 0.42
	2–3 m	21	8.02 ± 0.03	13.67 ± 0.13
		18	8.14 ± 0.04	14.72 ± 0.22
2015	Surface (0–0.7 m)	20	8.3 ± 0.22	6.28 ± 0.05
		20	8 ± 0.14	6.45 ± 0.04
	0–2 m	19	7.37 ± 0.15	6.44 ± 0.05
		19	10.42 ± 0.24	7.02 ± 0.05
	0–3 m	19	9.68 ± 0.29	6.71 ± 0.07
		16	10.13 ± 0.25	6.98 ± 0.06
	2–3 m	20	8.85 ± 0.22	6.59 ± 0.05
		20	8.8 ± 0.21	6.71 ± 0.05

### Experimental site and conditions

**Figure 1:**
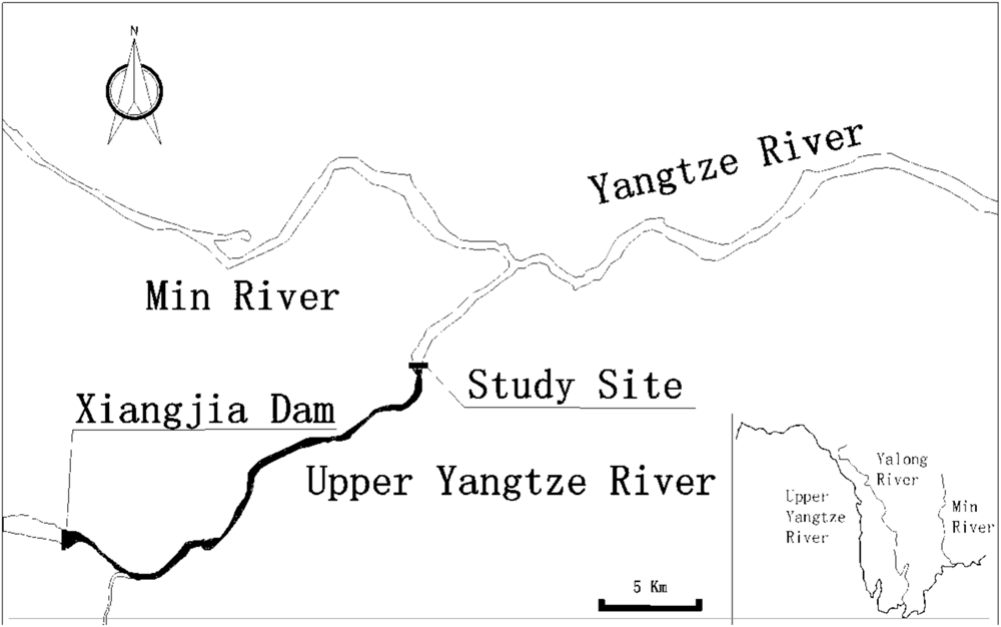
Study site located in upper Yangtze River and 24 km downstream of Xiangjiaba Dam, with only one tributary between Xiangjiaba Dam and the experimental site.

The experimental net cage system was installed at a position in the upper Yangtze River, 24 km downstream the Xiangjiaba Dam which was selected to illustrate that supersaturation persisted several kilometers from the dams. The location of the experimental barge by the river bank allowed a stable study site (moderate water-flow) with maximum water depth to perform the investigation. Fish observations were conducted from August 27 to September 2, 2014 and September 2 to September 10, 2015 (Fig. 1). Xiangjiaba Dam is the third-largest hydroelectric power station in China after the Three Gorges and Xiluodu Dams. In addition, there is only one tributary between Xiangjiaba Dam and the experimental site and this is not sufficient to reduce the TDGS levels downstream the confluence. The width of the river at our study site was 281 m and the water depth (>3 m) allowed installment of experimental cages.

### Experimental cages

The experimental setup differed slightly between 2014 and 2015 but in all cases net cages contained two duplicated fish groups, which were separated by a net screen.

**Figure 2:**
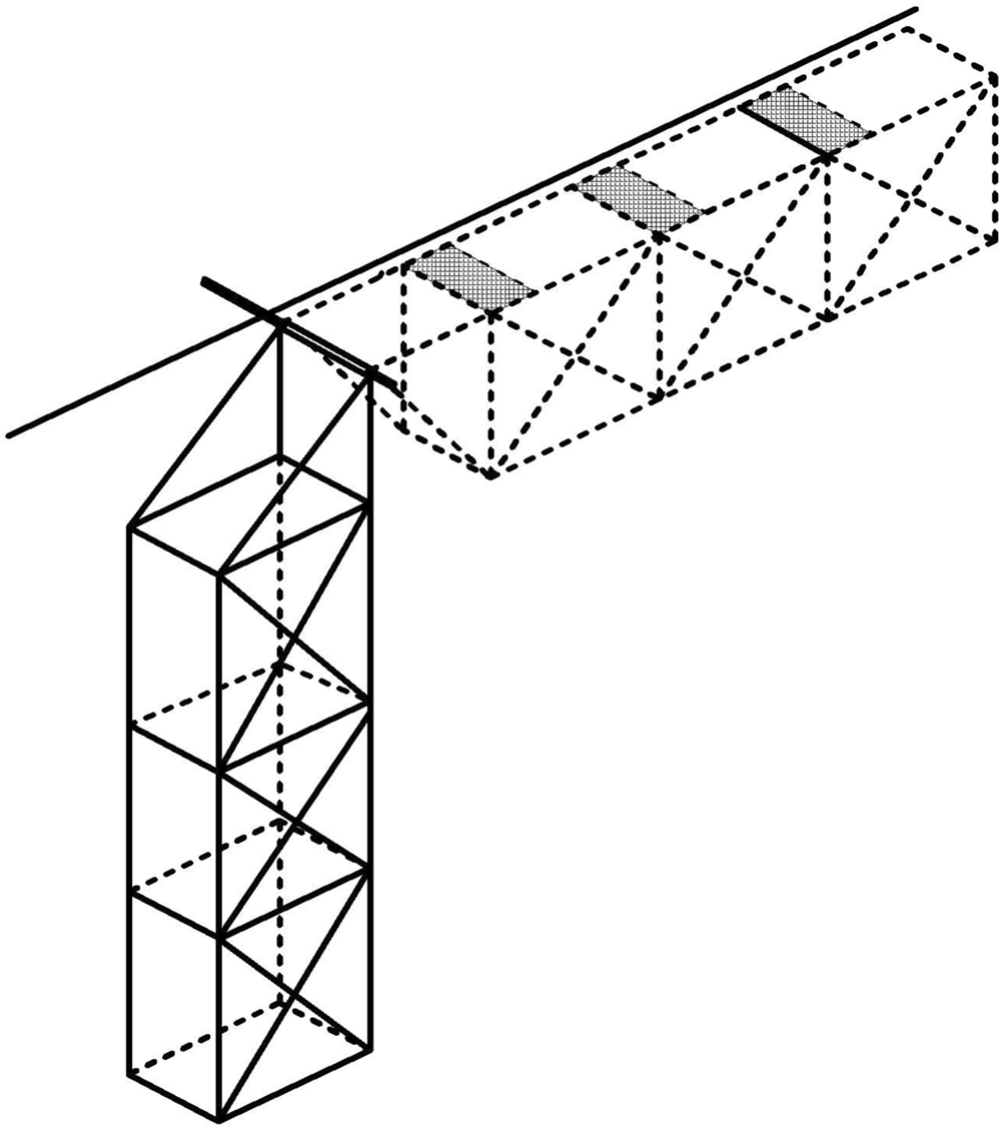
The 2014 experimental multilayer cage was divided into three compartments with each layer opening through the gray part when the multilayer cage revolved to a horizontal position around a fixed axle.
  The surface cage was found relatively limited in 2014 so in order to secure a study with more space per fish we used bigger tanks in the 2015 study. Accordingly, for the multilayer cage, we improved the experiment by providing fish access to a greater volume and water column (surface to a certain depth) as described below:
  - A total of 40 (2 × 20) fish were placed in two surface net pens (0.5 m × 1 m × 0.7 m (height)) allowing fish to swim to 0.7 m water depth.
  - Two multilayer cage systems were used. One with a depth allowing fish to swim freely in a 3 m water column (0–3 m) and the other one was divided into two compartments allowing fish to stay at depths of 0–2 m and 2–3 m, respectively (Table 1).

In 2014, two experimental cage systems were applied:

- Six cylindrical surface cages (height 0.25 m and diameter 0.7 m), each containing 10 fish were placed in the surface (0–0.3 m) of the river whereby fish were kept not deeper than 30 cm from the river surface.
- A multilayer cage system (0.8 m × 0.6 m × 3 m) was divided into three compartments (each containing two duplicated fish groups) corresponding to each depth (Table 1). The multilayer cage (Fig. 2) could be revolved around a fixed axle. It was kept in a vertical position during exposure to various depths but was turned to a horizontal position at the surface when fish in the different compartments were examined for GBD signs and sampled.

### TDGS, water velocity and temperature recording

During the observation period a Point Four Tracker (Point Four Systems Inc., Canada, measurement accuracy: 1%) for measurement of TDGS (%) in the river water was placed at the barge position within 1 m distance from the cages and data obtained every 2 h during daytime as well as twice at night in 2014 and four times per day in 2015. The current (water velocity) was measured at the same time points using a rotating current meter (Xiangruide Inc., China, measurement accuracy: 1.2%) in the surface layer of the water around the experimental site. A ZDR temperature recorder (Zheda Inc., China, Accuracy: ±0.5 °C) was placed at a 0.3 m water depth for continuous temperature recording throughout the study periods.

### Observation and sampling

Fish in the surface cages were visible and continuously observed. Fish in the multilayer cage compartments were observed when the multilayer cage was rotated to the horizontal position (every 2 h during daytime and twice during night in 2014 and four times every day in 2015). Each observation lasted 5 to 20 minutes. When the fish were displaying abnormal swimming behavior and balance disturbances (and considered dying) they were sampled and their clinical signs were recorded.

### Statistical analysis

Kaplan-Meier survival plot analysis was adopted to describe the survival time of fish kept under different conditions^22,23^ and the comparison of survival was analyzed with Log-rank test. Time to reach 50% mortality was expressed as the median lethal time (LT_50_) as described by Wang^24^ and one-way analysis of variance (ANOVA) was used to analyze the differences in mortality between cages. The significance level was set at *P* < 0.05 (Table 1).

## Results

**Figure 3:**
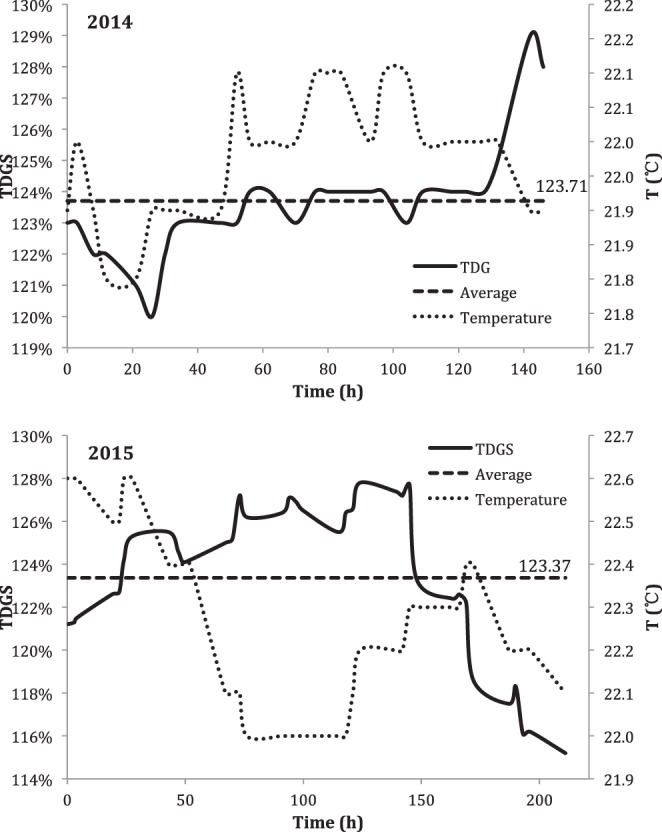
Variations of TDGS and temperature during the period of observation in 2014 and 2015 (the solid line is the variations of TDGS, the dotted line is the variations of temperature and the horizontal dashed line is the average of TDGS levels during the experimental period).

The TDGS level at the experimental site varied considerably over the observation period (7 days in 2014 and 9 days in 2015) (Fig. 3). In the 2014 observation period the TDGS level declined gradually from 122% to 120% the first day. Thereafter it slowly ascended to 124% (day 3) and stayed constant until the last day of the observation period, where the TDGS level sharply rose to 129%. The average TDGS level was 123% during the 7 days. In 2015, during the first 6 days, the TDGS level rose from 121% to 127% whereafter it fell rapidly until the end of the experiment with an average TDGS level at 123%. The average current (water velocity) was 0.11 m/s (max 0.3 m/s and min 0.02 m/s) in 2014 and 0.10 m/s (max 0.21 m/s and min 0.05 m/s) in 2015. The temperature was relatively stable and varied between 22.0 to 22.5 °C in 2014 and 22.0 to 22.6 °C in 2015.

**Figure 4:**
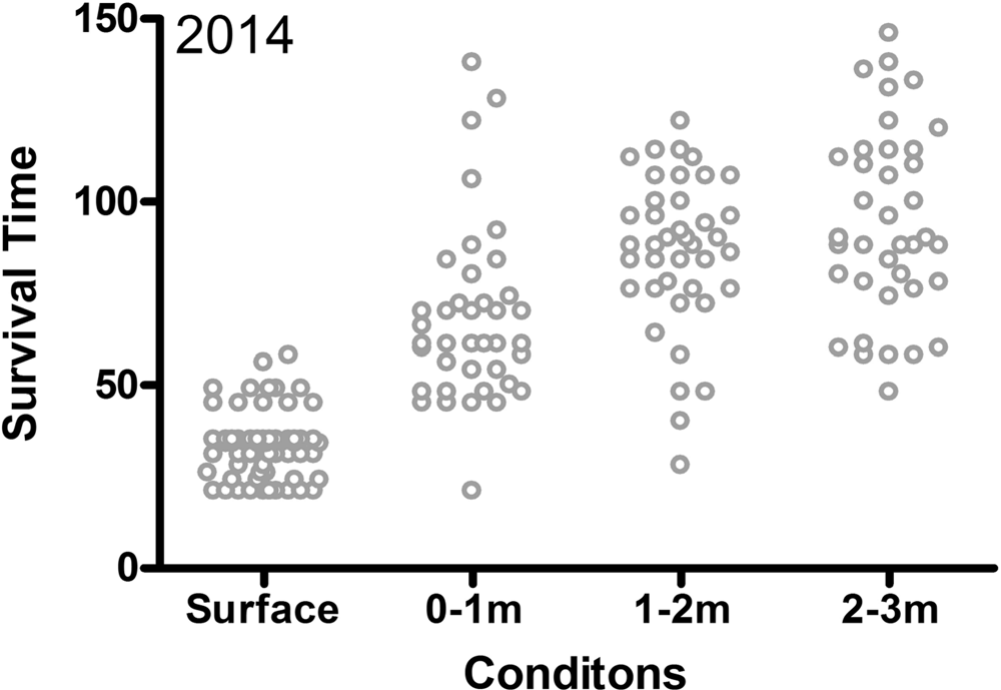
Survival time of fish kept at different water depths. Each dot represent the survival time of a fish.

**Figure 5:**
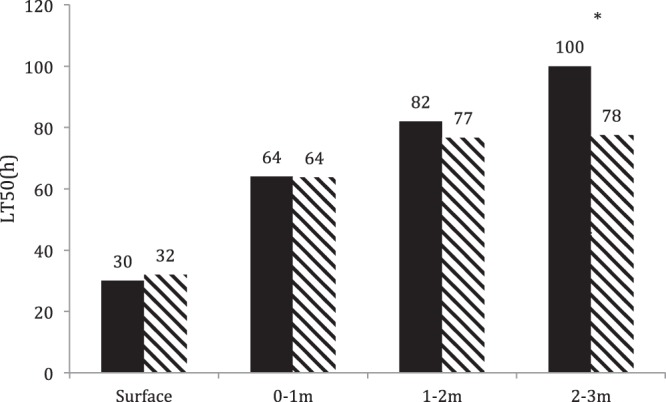
LT_50_ of fish groups at different water depths in 2014. Black column and zebra column represent the two groups, *significant difference, p < 0.05.

In 2014, all fish in all experimental groups reached the near-lethal stage within the experimental period and no significant differences between groups were found except for the fish kept at 2–3 m water depth. The survival time increased with depth. In surface cages all fish reached the lethal stage from 21 h to 60 h. In 0–1 m cages, 80% of fish died within the period 45–92 h, whereas fish survived up to 112 h in deeper cages (Fig. 4). The calculated LT_50_ (Fig. 5) increased from surface cages (0–0.3 m) to 2–3 m cages. LT_50_ in the 0–1 m cage was about double as that found in the surface cages (0–0.3 m), and the LT_50_ in the 2–3 m cage increased further when compared to the level in the 1–2 m cage. There was no significant difference between the depth of 1–2 m and 2–3 m.

In 2015, only the fish in the surface cage (0–0.7 m) exhibited 100% mortality and mortalities decreased with depth (68.42% and 52.63% mortality at 0–2 m, 42.11% and 6.25% mortality at 0–3 m and at 2–3 m depth mortalities reached 10% and 35%. LT_50_ was only calculated for surface (0–0.7 m) and 0–2 m groups in 2015 because the other groups did not reach 50% mortality. The LT50 in surface groups were 77 h and 83 h while in the groups of 0–2 m, the LT50 were 142 h and 151 h. No significant difference between groups at 0–3 m and 2–3 m was seen.

### Behaviour

**Figure 6:**
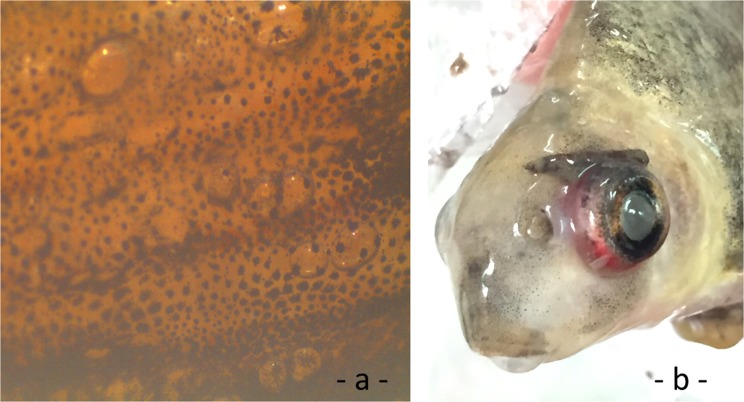
GBD signs of Chinese sucker (**a** bubbles in fins; **b** exophthalmia).

Due to the turbid water, the behavior of fish was only observed in the surface cages in 2014. No clear behavioral changes were observed in the first 10 h of exposure, but thereafter fish displayed balance disturbances (whirling) or showed ascending and descending movements. GBD affected fish exhibited exophthalmia and/or gas bubbles distributed in all fins associated with capillary swelling and bleeding (Fig. 6).

**Figure 7:**
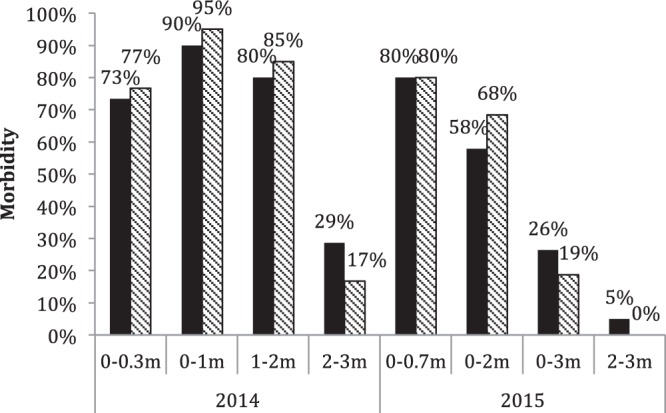
Morbidity under different conditions in 2014 and 2015, black column and zebra column represent the two groups kept at the same condition.

Occurrence of GBD in the different experimental groups varied (Fig. 7). Except from fish kept at the surface (0–0.3 m) in 2014 a clear trend for a decrease of GBD with water depth was noted.

## Discussion

The present study indicated that water depth is associated with decreased development of GBD in fish exposed to supersaturated water. The increased hydrostatic pressure at deeper positions could explain the observations. However, influence of other parameters, such as variations of temperature and velocity in different water depths, should be considered as these factors may affect fish bioenergetics and behavior^25,26^. We therefore evaluated variations in the water column but found no differences of temperature and velocity in the upper 0–3 m surface layers. However, as water velocity and temperature are decisive elements affecting fish physiology and behavior we recommend to include additional study sites, where velocity and temperature may vary, in future investigations in order to reach a full picture of parameters affecting fish health in rivers influenced by dam constructions.

Tolerance to TDGS differs between fish species^14,27,28^, and it is important to characterize the GBD susceptibility for each individual fish species. The present study showed that Chinese sucker is sensitive to TDGS at average levels around 123%. In 2014, all Chinese suckers died during the experimental period whereas, only the fish in the surface cages (0–0.7 m) exhibited 100% mortality in 2015. The water depth had an effect on GBD development. Even when 100% mortality was observed, as in 2014, a delayed disease development was noted with water depth. Fish in the surface cages died within 2.5 days, whereas the median lethal time periods were delayed to 5 days and 7 days at the 1–2 m and 2–3 m cages, respectively. Correspondingly, the LT_50_ increased from 33.19 h in the surface cage to 67.79 h in 0–1 m cage, 85.24 h in the 1–2 m cage and 92.85 h in the 2–3 m cage. This was further framed in the 2015 study where mortality decreased from 100% in surface cage (0–0.7 m) to 42.11% and 6.25% in 0–3 m cages. In addition, the LT_50_ increased from 77.43 h and 82.89 h in surface cage (0–0.7 m) to 142.22 h and 150.99 h in 0–2 m cages. This corroborates observations by Heggberget^14^ showing that GBD signs ameliorate with water depth. This author kept brown trout, *Salmo truttta*, European eel, *Anguilla Anguilla* and perch, *Perca* in supersaturated water at 0 and 3 m depths and subsequently it was observed that almost all surface fish died, whereas no mortalities were observed at 3 m depth.

The decrease of the severity of GBD symptoms indicated the ameliorating effect of depth when fish were exposed to supersaturated water. GBD morbidity of Chinese sucker decreased with water depth both in 2014 and 2015. Thus, 90% and 95% fish showed GBD in 0–1 m cages but only 29% and 17% were affected at 2–3 m depths in 2014. In 2015, 80% surface fish were GBD affected whereas only 0% and 5% fish showed GBD signs at 2–3 m depth. Correspondingly, a range of North American fish species (large scale sucker, *Catostomus macrocheilus*, long nose sucker, *Catostomus catostomus*, northern pike-minnow, *Ptychocheilus oregonensis*, red side shiner, *Richardsonius balteatus* and walleye, *Sander vitreus*), which were kept in supersaturated water at deeper water, was found to develop GBD at lower frequency compared to surface fish^17^. The physical explanation may be found in Henry’s law, stating that the amount of dissolved gas is proportional to its partial pressure in the gas phase. Deep water layers with higher hydrostatic pressure dissolve more gas, and correspondingly Weitkamp and Katz (1980)^29^ found that for each meter of increasing depth the pressure will compensate for approximately 10% of the TDGS. When the barometric pressure plus the hydrostatic pressure is equal to the TDGS in the surface water, the water depth is termed the hydrostatic compensation depth. Some fish may compensate for adverse effects by regulation of swimming depth and direction^17–19,30^ and theoretically fish would not contract GBD if constantly staying under the hydrostatic compensation depth. Hence, for the 123% to 124% TDGS levels, GBD should not typically occur below the compensation depth 2.3 m to 2.4 m. However, in our experiments, fish exposed below the compensation depth still showed the signs of GBD. Such a deviation from theoretical calculations was also seen by Weitkamp^8^ and Ryan^31^ who collected resident fish from deeper water and found that some of them were affected by GBD. This may partly be explained by the behavior of fish. If fish have inefficient avoidance behavior and if they are not able move to deeper water with high hydrostatic pressure, GBD may still develop. Juvenile Chinese suckers usually occupy surface water and laboratory investigations have demonstrated that the Chinese sucker only partly avoid highly supersaturated water^32^. The slightly different observations during the 2014 and 2015 studies may be explained by the different sampling methods and experimental set-up. The sampling method in 2014 was associated with a longer duration of contact/observation and more frequent sampling. Thus, when sampled fish are taken from the depth and translocated to low pressure conditions at the surface, where the observer is placed, it is expected that GBD signs will develop before or later. The present study was conducted at a river site with relatively shallow water and the physico-chemical parameters, except for the hydrostatic pressure, were found to be uniform in the water column. Thus, temperature, current and supersaturation values were the same at all depths minimizing effects on these parameters on the experiment.

In conclusion, when applying Chinese sucker as an experimental model we found that exposure to TDGS is associated with development of GBD and mortality. It was shown that Chinese sucker succumbed within a few days post-exposure when forced to live in shallow water with TDGS levels above 120%. We also demonstrated that increased water depths may alleviate the disease symptoms and reduce mortality and suggest that increased hydrostatic pressure in deeper water layers will explain the observations by Henry’s law. Thus, a compensation depth of 3 m can delay some of the adverse reactions but fish in shallow waters are more prone to GBD. TDGS is mainly caused by dam construction and discharge during the flooding season. As these are common in the Yangtze River, which is the habitat of many fish species including Chinese sucker, fish in this river will be at risk if their escape behavior is poorly developed. Assessment of individual fish species and their tolerance to TDGS should therefore be performed. In addition, it should be considered to modify dam operations in order to lower the TDGS level and/or shorten discharge periods. Our observations also suggest that securing high water depths downstream the dam may be a possible way to counteract fish morbidity and mortality. Further, additional technological solutions, such as trickling filters, for desaturation of the river water may be considered.

## References

[1] Williams JG (2008). Mitigating the effects of high-head dams on the Columbia River, USA: experience from the trenches. Hydrobiologia.

[2] Williams JG, Smith SG, Muir WD (2001). Survival estimates for downstream migrant yearling juvenile salmonids through the Snake and Columbia rivers hydropower system, 1966–1980 and 1993–1999. North American Journal of Fisheries Management.

[3] Gale WL, Maule AG, Postera A, Peters MH (2004). Acute exposure to gas‐supersaturated water does not affect reproductive success of female adult chinook salmon late in maturation. River Research and Applications.

[4] Feng J, Li R, Liang RF, Shen X (2014). Eco-environmentally friendly operational regulation: an effective strategy to diminish the TDG supersaturation of reservoirs. Hydrology and Earth System Sciences.

[5] Crunkilton RL, Czarnezki JM, Trial L (1980). Severe gas bubble disease in a warmwater fishery in the midwestern United States. Transactions of America Fisheries Society.

[6] Donna SL (1995). Gas Supersaturation and Gas Bubble Trauma in Fish Downstream from a Midwestern Reservoir. Transaction of America Fisheries society.

[7] Ebel WJ (1969). Supersaturation of nitrogen in the Columbia River and its effect on salmon and steelhead trout. Fishery Bulletin.

[8] Weitkamp DE, Sullivan RD, Swant T, DosSantos J (2003). Gas bubble disease in resident fish of the lower Clark Fork River. Transactions of the American Fisheries Society..

[9] Nunn J, Fidler L, Northcott P (1993). Investigation of changes to the operation of Keenleyside Dam to reduce supersaturation of dissolved gases downstream. CSCE Annual Conference.

[10] Qu L, Li R, Li J, Li KF, Deng Y (2011). Field observation of total dissolved gas supersaturation of high-dams. Science China Technological Sciences.

[11] Li R (2009). Prediction for supersaturated total dissolved gas in h igh-dam hydropower projects. Sci China Ser E-Tech Sci.

[12] Wang, Y. M., Li, Y., An, R. D. & Li, K. F. Effects of total dissolved gas supersaturation on the swimming performance of two endemic fish species in the upper yangtze river. *Scientific Reports*, **8** (2018). 10.1038/s41598-018-28360-7 PMC6030173 29968818

[13] Gerald RB (1980). Etiology of Gas Bubble Disease. Transaction of America Fisheries society.

[14] Heggberget TG (1984). Effect of supersaturated water on fish in the River Nidelva, southern Norway. Journal of fish biology.

[15] Ebel WJ, Raymond HL (1976). Effect of atmospheric gas supersaturation on salmon and steelhead trout of the Snake and Columbia rivers. Marine Fisheries Review.

[16] CCTV (China Central Television). Shichuan Jinsha salvage 40 tons ofdead fish poisoning may exclude official, http://news.cntv.cn/2014/07/19/ARTI1405750580177886.shtml (2014)

[17] Beeman JW, Maule AG (2006). Migration depths of juvenile Chinook salmon and steelhead relative to total dissolved gas supersaturation in a Columbia River reservoir. Transactions of the American Fisheries Society.

[18] Johnson EL (2010). Migration depths of adult steelhead Oncorhynchus mykiss in relation to dissolved gas supersaturation in a regulated river system. Journal of fish biology.

[19] Knittel M, Chapman G, Garton R (1980). Effects of hydrostatic pressure on steelhead survival in air-supersaturated water. American Fisheries Society.

[20] Gao Z, Li Y, Wang W (2008). Threatened fishes of the world: Myxocyprinus asiaticus Bleeker 1864 (Catostomidae). Environmental Biology of Fishes.

[21] Zhang CG, Zhao YH, Kang JG (2000). A discussion on resources status of Myxocryprinus asiaticus (Bleeker) and their conservation and the recovery. Journal of Natural Resources.

[22] Allison, P. D. Survival analysis using SAS: a practical guide. Cary NC: Sas Institute (1995).

[23] Rao P (2000). Applied survival analysis: regression modeling of time to event data. Journal of the American Statistical Association.

[24] Wang YM, Li KF, Li J, Li R, Deng Y (2015). Tolerance and Avoidance Characteristics of Prenant’s Schizothoracin Schizothorax prenanti to Total Dissolved Gas Supersaturated Water. North American Journal of Fisheries Management.

[25] Crowder DW, Diplas P (2000). Evaluating spatially explicit metrics of stream energy gradients using hydrodynamic model simulations. Canadian Journal of Fisheries and Aquatic Sciences.

[26] Gualtieri C., Ianniruberto M., Filizola N., Santos R., Endreny T. (2017). Hydraulic complexity at a large river confluence in the Amazon basin. Ecohydrology.

[27] Chen SC (2012). Effects of total dissolved gas supersaturated water on lethality and catalase activity of Chinese sucker (Myxocyprinus asiaticus Bleeker). Journal of Zhejiang University Science B.

[28] Huang X, Li KF, Du J, Li R (2010). Effects of gas supersaturation on lethality and avoidance responses in juvenile rock carp (Procypris rabaudi Tchang). Journal of Zhejiang University Science B.

[29] Weitkamp DE, Katz M (1980). A review of dissolved gas supersaturation literature. Transactions of the American Fisheries Society.

[30] Johnson EL (2007). Estimating adult Chinook salmon exposure to dissolved gas supersaturation downstream of hydroelectric dams using telemetry and hydrodynamic models. River research and applications.

[31] Ryan BA, Dawley EM, Nelson RA (2000). Modeling the effects of supersaturated dissolved gas on resident aquatic biota in the main-stem Snake and Columbia Rivers. North American Journal of Fisheries Management.

[32] Cao L (2015). The tolerance threshold of Chinese sucker to total dissolved gas supersaturation. Aquaculture Research.

